# Epidemiological Trends and Shared Molecular Signatures of Pancreatic Ductal Adenocarcinoma and Diabetes: An Integrative Analysis Based on Global Burden of Disease and Gene Expression Omnibus Datasets

**DOI:** 10.7150/jca.134528

**Published:** 2026-05-25

**Authors:** Jiaxi Zhang, Zhibin Liu, Ke Huang, Junkoo Yi, Myoung Ok Kim

**Affiliations:** 1 Department of Animal Science and Biotechnology, Research Institute for Innovative Animal Science, Kyungpook National University, Sangju 37224, Republic of Korea.; 2 School of Animal Life Convergence Science, Hankyong National University, Anseong, Republic of Korea.; 3 Gyeonggi Regional Research Center, Hankyong National University, Anseong, Republic of Korea.

**Keywords:** pancreatic ductal adenocarcinoma, diabetes mellitus, global burden of disease, gene expression omnibus, integrative analysis

## Abstract

**Background:**

Pancreatic ductal adenocarcinoma (PDAC) is one of the most lethal malignancies worldwide, and diabetes mellitus has been recognized as both a risk factor for and a potential consequence of PDAC. However, the epidemiological trends and shared molecular mechanisms underlying this association are not completely understood.

**Methods:**

We conducted an integrative analysis combining population-level epidemiological data and transcriptomic datasets. Global trends in PDAC incidence, PDAC-related mortality, and diabetes incidence from 2013 to 2023 were obtained from the Global Burden of Disease (GBD) database. A meta-analysis was performed to quantify the association between diabetes and PDAC risk. In addition, gene expression datasets focusing on PDAC (GSE15471) and type 2 diabetes mellitus (T2DM; GSE20966) were obtained from the Gene Expression Omnibus database, differentially expressed genes were identified, and upregulated genes associated with both PDAC and T2DM were examined using heatmaps.

**Results:**

Continuous increases in global PDAC incidence, mortality due to PDAC, and the incidence of diabetes were identified in the GBD data. The meta-analysis of eight studies demonstrated that diabetes was significantly associated with an increased risk of PDAC (pooled relative risk = 1.94, 95% CI: 1.78-2.11). Transcriptomic analyses identified five genes—*FXYD3*, *LAMC1*, *MDFIC*, *SOCS3*, and *TREM2*—that were consistently upregulated in both PDAC and T2DM-affected tissues.

**Conclusion:**

This integrative epidemiological and transcriptomic analysis demonstrates that diabetes and PDAC share not only similar global disease trends but also a significant epidemiological association and common molecular alterations. The identified shared genes may represent molecular links between diabetes and PDAC, providing new insights applicable to risk stratification and mechanistic studies.

## 1. Introduction

Pancreatic ductal adenocarcinoma (PDAC) the most common malignant form of pancreatic cancer, exhibits highly aggressive biological behavior, resulting in exceptionally poor prognoses 1. In recent years, PDAC incidence and mortality due to PDAC have both continued to rise, making it one of the most pressing challenges in global public health. According to GLOBOCAN 2020, approximately 496,000 new cases and 466,000 deaths occur annually worldwide, reflecting an almost one-to-one incidence-mortality ratio and underscoring PDAC's extreme lethality. Incidence varies considerably by region, with North America, Europe, Australia, and parts of East Asia reporting the highest disease burdens. Although incidence remains relatively low in low-income regions, the burden is increasing due to population aging, urbanization, and lifestyle changes 2. Forecasts indicate that, if current trends persist, global deaths due to PDAC will exceed 800,000 by 2040, ranking it among the leading causes of cancer-related death 3.

Risk factors for PDAC fall broadly into genetic, lifestyle, and disease-related categories 4. Regarding genetic factors, approximately 5-10% of patients have a family history, and mutations in relevant pathogenic genes can significantly increase the risk. Among lifestyle factors, long-term cigarette smoking is one of the strongest modifiable determinants, more than doubling PDAC risk 5. Obesity and metabolic syndrome contribute through chronic inflammation, insulin resistance, and lipid metabolism dysregulation. Alcohol intake, chronic pancreatitis, and the long-term consumption of a high-fat diet are similarly associated with elevated PDAC risk 6.

Epidemiological and clinical data indicate that type 2 diabetes mellitus (T2DM) is a key risk factor for PDAC, with numerous studies showing that long-standing diabetes increases PDAC risk approximately 1.5-2-fold 7. The development of new-onset diabetes demonstrates a strong temporal association with tumor development, particularly in adults over 50, and is increasingly recognized as a potential early indicator of occult malignancy 8. Importantly, this relationship is bidirectional: while diabetes promotes oncogenesis through chronic hyperglycemia, hyperinsulinemia, and systemic inflammatory activation, PDAC itself can induce a distinct paraneoplastic form of diabetes (type 3c diabetes) via β-cell dysfunction, impaired insulin signaling, and metabolic derangement 9, 10. While these interconnected metabolic and inflammatory processes help explain how diabetes can be both a risk factor for and consequence of PDAC, our understanding of the complex biological basis underlying this bidirectional relationship remains incomplete.

Although numerous studies have explored the association, most have focused on either epidemiological correlations or isolated molecular mechanisms 11, 12. Global-level analyses describing long-term trends in PDAC and diabetes are relatively limited, and transcriptomic studies rarely compare gene expression alterations across both diseases in a systematic manner 13, 14. As a result, population-level disease burden and molecular-level evidence have not been sufficiently integrated.

The present study aimed to integrate epidemiological and transcriptomic data to comprehensively investigate the relationship between diabetes and PDAC. Analyzing Global Burden of Disease (GBD) data, we examined recent global trends in PDAC incidence, PDAC mortality, and diabetes incidence. In parallel, we analyzed publicly available gene expression datasets from the Gene Expression Omnibus (GEO) database to identify upregulated genes shared between PDAC and T2DM. This integrative approach provides multi-level evidence linking diabetes and PDAC and may help uncover molecular signatures underlying their association.

## 2. Materials and Methods

This study employed an integrative analytical strategy combining epidemiological and transcriptomic analyses to investigate the association between PDAC and diabetes mellitus. All analyses were conducted using RStudio (R version 4.5.2).

### 2.1 GBD analysis

Epidemiological data were obtained from the GBD database using the GBD Results Tool. Data from 2013 to 2023 were extracted for pancreatic cancer incidence, pancreatic cancer mortality, and diabetes incidence. Annual trends were summarized and visualized to evaluate temporal changes in PDAC burden and diabetes incidence at the global level. Because given that PDAC accounts for more than 90% of all pancreatic cancer cases, pancreatic cancer indicators from the GBD database were used as a proxy for PDAC in this study, consistent with previous epidemiological analyses.

### 2.2 Meta-analysis of published studies

To further assess the epidemiological association between diabetes mellitus and PDAC, we performed a meta-analysis of previously published studies. Eight eligible studies reporting risk estimates (relative risk [RR]) and their corresponding 95% confidence intervals (CIs) were included 9, 15-21. Relevant data, such as first author, publication year, and effect estimate, were extracted from each study.

The pooled effect size was calculated using a random-effects model, with the Hartung-Knapp correction used to control for potential variability among studies. Statistical heterogeneity was assessed using the *I*² statistic, τ² value, and Cochran's *Q* test. A two-sided *p*-value < 0.05 was considered statistically significant.

### 2.3 GEO data acquisition and processing

Gene expression datasets were retrieved from the GEO database: dataset GSE15471 was used to analyze gene expression differences between PDAC-affected and normal pancreatic tissues, and dataset GSE20966 was used to analyze gene expression differences between pancreatic tissue from individuals with T2DM and pancreatic tissue from non-diabetic control individuals. Only samples with clear diagnostic classifications were included.

### 2.4 Differential expression and shared gene identification

After processing and normalization, differential expression analyses were performed separately for the PDAC and T2DM gene expression datasets using the *limma* package. As no direct integration or merging of expression matrices was conducted across datasets, batch effect correction was not applied. This strategy minimizes potential batch-related bias between datasets while preserving dataset-specific biological signals. Genes with an adjusted *p*-value < 0.05 and |log₂ fold change| > 1 were considered to exhibit significant differential expression. Genes that were upregulated or downregulated in both datasets were identified as shared upregulated and downregulated genes.

### 2.5 Visualization

Heatmaps were generated to visualize the expression patterns of shared upregulated genes in the disease and control groups.

## 3. Results

### 3.1 Geographical differences in PDAC incidence and mortality due to PDAC

**Figure 1:**
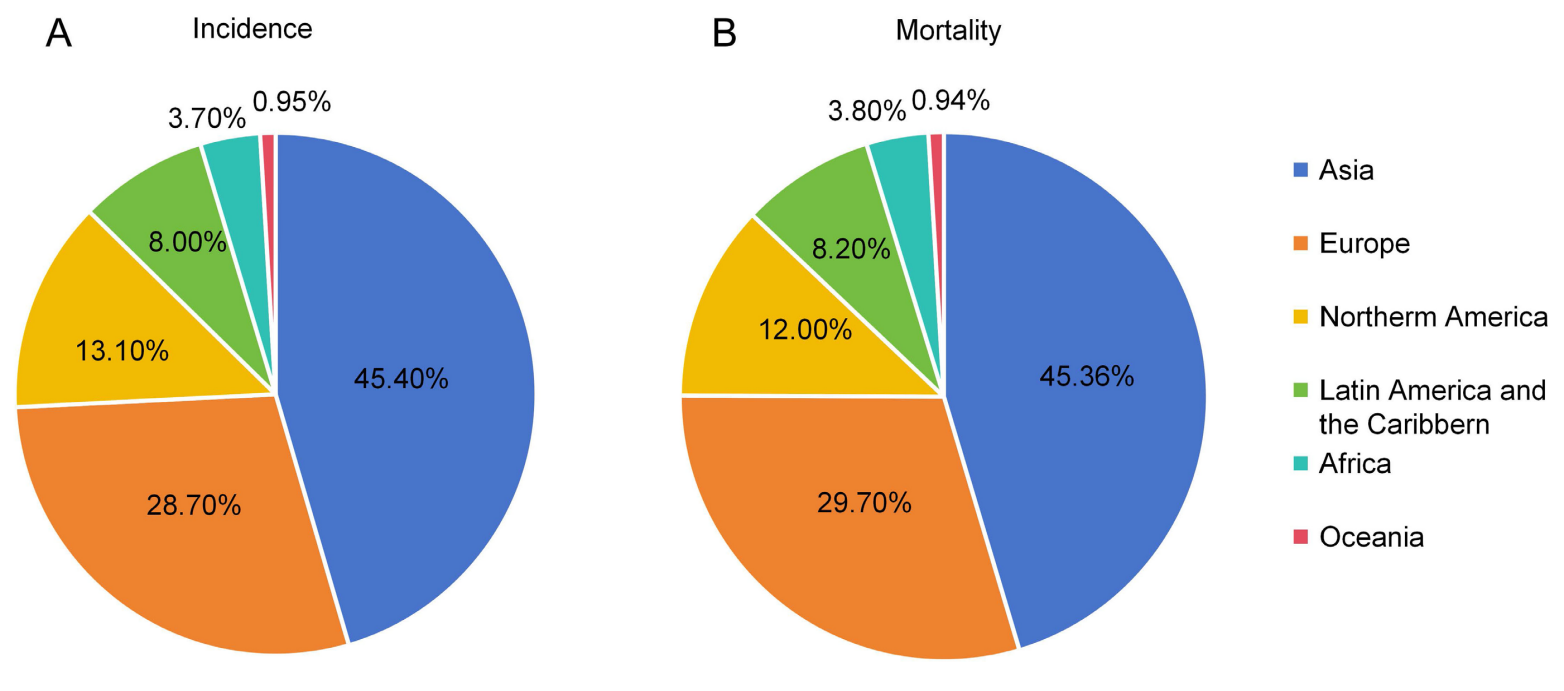
Global PDAC incidence (A) and mortality (B) by region based on GLOBOCAN 2020 data.

Based on data from GLOBOCAN 2020, we analyzed the global distribution of PDAC incidence and PDAC-related mortality by region (Figure 1). Asia accounted for the largest proportion of global PDAC incidence (approximately 45.4%), followed by Europe (28.7%) and North America (13.1%) (Figure 1A). In contrast, Latin America and the Caribbean (8.0%), Africa (3.7%), and Oceania (< 1%) contributed relatively small proportions.

A similar geographical pattern was observed for PDAC-related mortality (Figure 1B). Asia represented the highest proportion of global PDAC-related deaths (approximately 45.3%), followed by Europe (29.7%) and North America (12.0%), while proportions in Latin America and the Caribbean (8.2%), Africa (3.8%), and Oceania (< 1%) remained comparatively low.

Taken together, these results demonstrate marked geographical disparities in the global burden of PDAC, with incidences and mortalities concentrated in Asia, Europe, and North America, while Africa, Latin America, and Oceania exhibited relatively low disease burdens. This suggests substantial regional heterogeneity in the global epidemiology of PDAC.

### 3.2 Global trends of PDAC and diabetes based on GBD data

**Figure 2:**
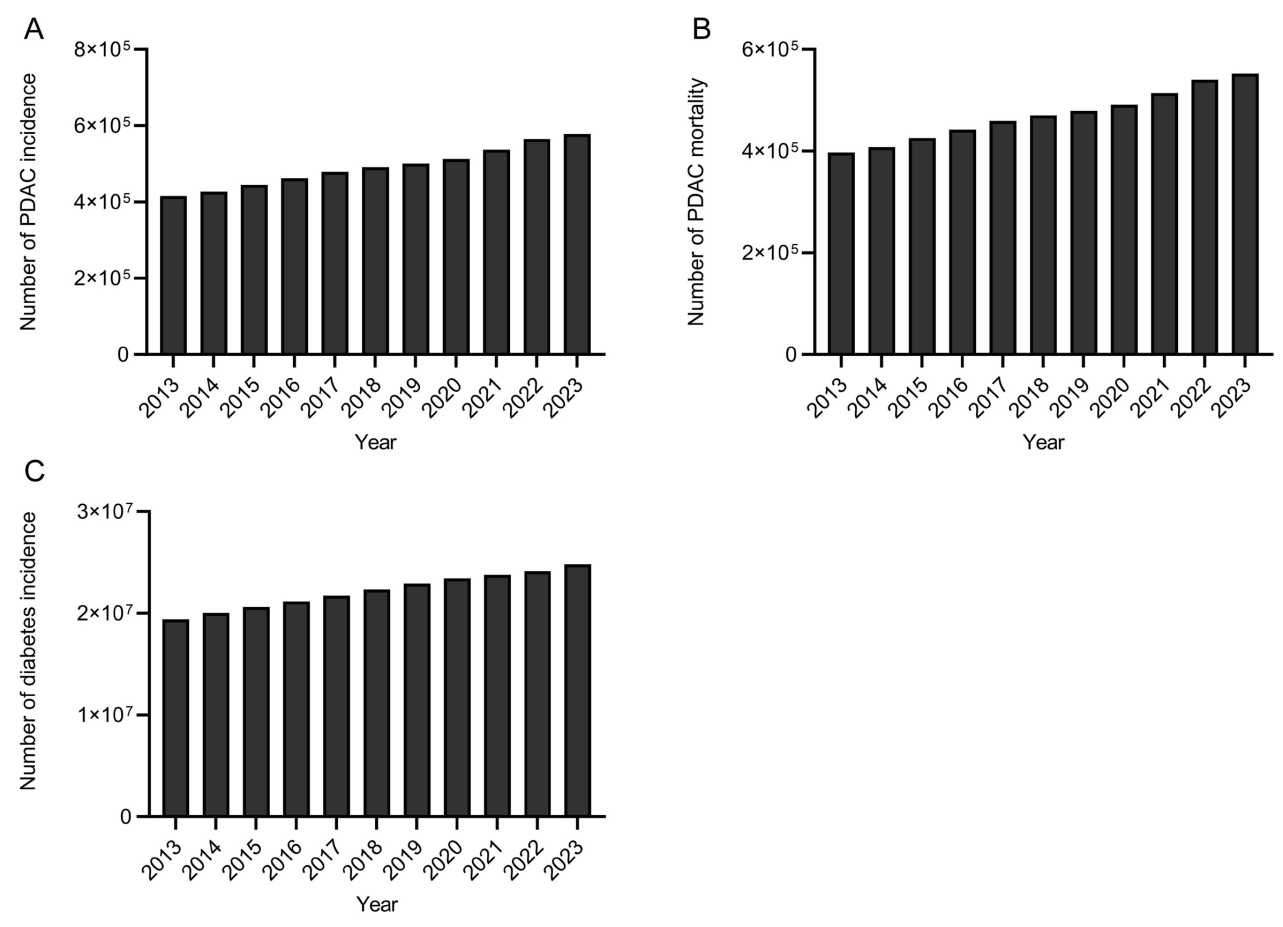
Global trends in PDAC incidence (A), PDAC-related mortality (B), and diabetes incidence (C) from 2013 to 2023 based on GBD data. PDAC incidence is expressed as the annual number of newly diagnosed cases, PDAC-related mortality is expressed as the annual number of PDAC-related deaths, and diabetes mellitus incidence is expressed as the annual number of individuals living with diabetes.

Using GBD data, we analyzed temporal trends in the global incidence of and mortality due to PDAC as well as the incidence of diabetes from 2013 to 2023 (Figure 2).

As shown in Figure 2A, the global incidence of PDAC increased steadily during the analyzed period, with the annual number of newly diagnosed cases rising from approximately 4.1 × 10⁵ in 2013 to over 5.7 × 10⁵ in 2023, indicating a continuously increasing global disease burden. Similarly, PDAC-related mortality exhibited a persistent upward trend (Figure 2B), with the annual number of deaths increasing from approximately 3.9 × 10⁵ to approximately 5.5 × 10⁵ over the same period. This consistent annual increase in deaths reflects the sustained and increasing fatalities associated with this disease burden worldwide.

In parallel, global trends in diabetes incidence also showed a consistent upward trend (Figure 2C). The total number of individuals living with diabetes increased from approximately 1.9 × 10^7^ in 2013 to more than 2.6 × 10^7^ in 2023, highlighting a substantial and growing global diabetes burden.

Taken together, the parallel increasing trends in global pancreatic cancer incidence, mortality, and diabetes incidence suggest a possible temporal correlation between these two diseases.

### 3.3 Association between diabetes and PDAC: meta-analysis results

**Figure 3:**
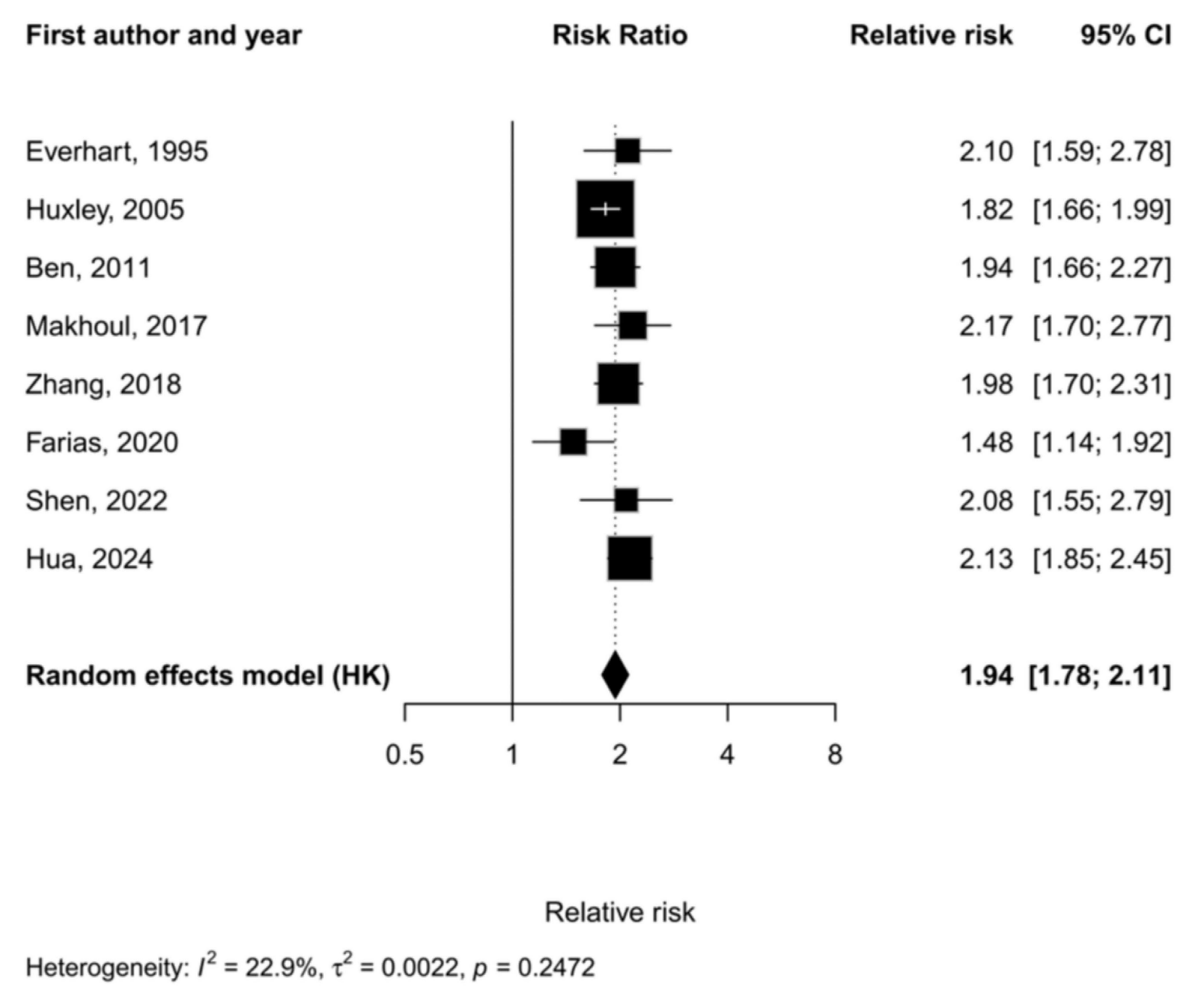
** Forest plot showing the association between diabetes and PDAC.** Squares represent study-specific estimates, with error bars indicating the 95% confidence interval, and the diamond represents the pooled effect calculated using the Hartung-Knapp (HK) random-effects model. Inter-study heterogeneity statistics for the meta-analysis are included at the bottom left. Statistical heterogeneity was assessed using the I² statistic, τ² value, and Cochran's Q test. A two-sided p-value < 0.05 was considered statistically significant.

This meta-analysis included eight published studies, all of which reported an increased risk of PDAC in patients with diabetes, with relative risks ranging from 1.48 to 2.17. Under the Hartung-Knapp random-effects model, the pooled relative risk, 1.94 (95% CI: 1.78-2.11), was significant, indicating that the risk of PDAC in diabetic patients is almost twice that of non-diabetic patients (Figure 3). Inter-study heterogeneity was low (*I*² = 22.9%, τ² = 0.0022), and Cochran's *Q* test returned a nonsignificant result (*p* = 0.247), indicating relatively consistent findings among the included studies.

### 3.4 Upregulated genes shared between PDAC and T2DM

**Figure 4:**
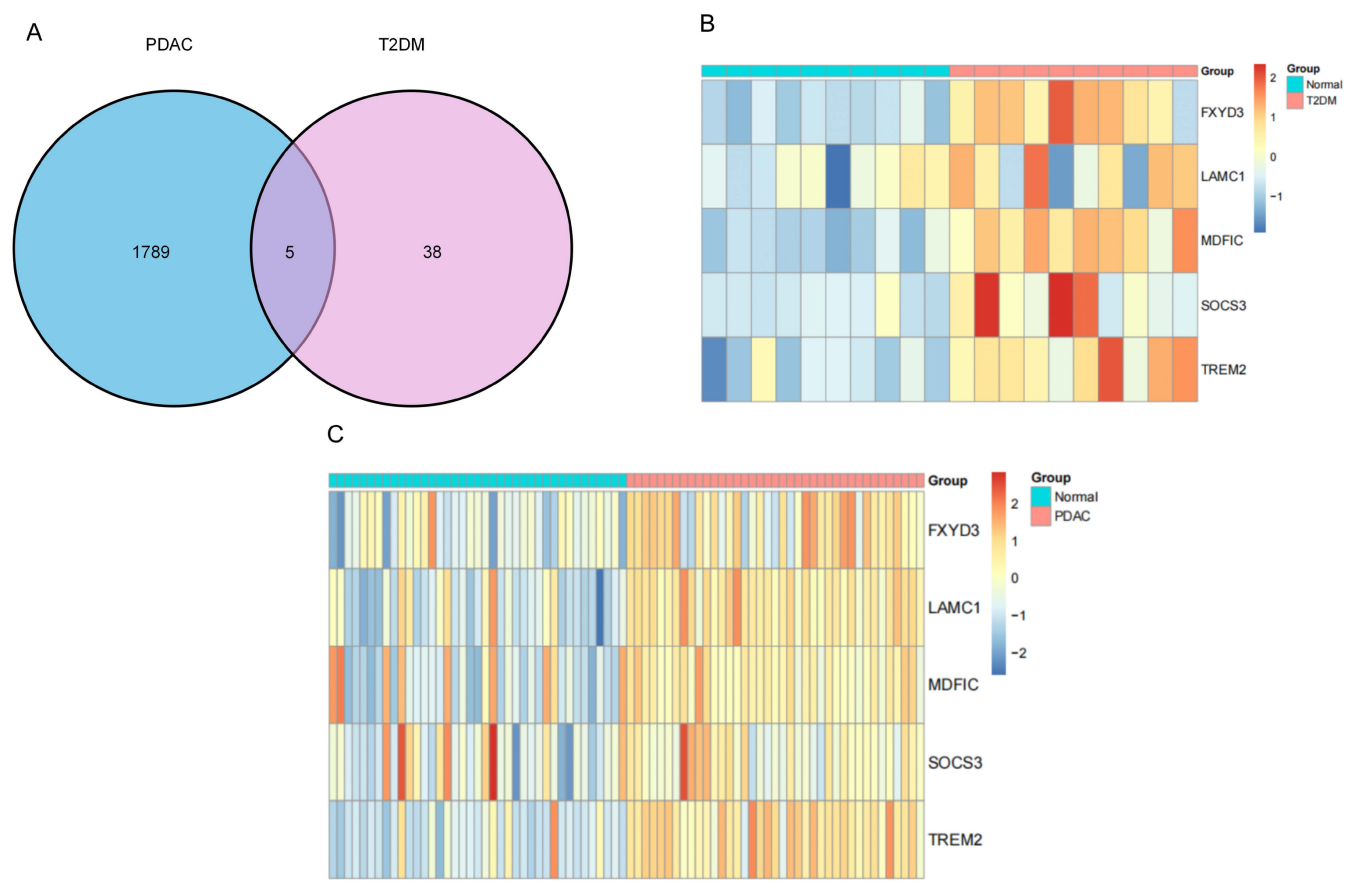
** Identification of upregulated genes shared between PDAC and T2DM and their expression patterns.** (A) A Venn diagram shows the overlap of significantly upregulated genes identified in the PDAC and T2DM datasets. (B, C) Heatmaps illustrate the expression patterns of the five shared upregulated genes, *FXYD3*, *LAMC1*, *MDFIC*, *SOCS3*, and *TREM2*, in PDAC-affected and normal pancreatic tissues (B) and in individuals with T2DM and in non-diabetic control individuals (C).

Given the close epidemiological association between PDAC and T2DM, we investigated whether the two diseases share common transcriptomic alterations. To this end, we analyzed the gene expression profiles in the GSE15471 and GSE20966 datasets to identify genes associated with PDAC and T2DM (Supplement Table 1), respectively, and explore their potential overlap (Figure 4).

By comparing PDAC-affected and normal pancreatic tissues, our differential expression analysis identified 1,789 upregulated genes associated with PDAC, and when compared with those from non-diabetic individuals, 38 genes were upregulated in pancreatic tissues from T2DM sufferers (Figure 4A). Analyzing the intersection between these upregulated gene sets revealed five shared genes, namely *FXYD3*, *LAMC1*, *MDFIC*, *SOCS3*, and *TREM2*. We also examined commonly downregulated genes between the PDAC and T2DM datasets. However, no overlapping downregulated genes were identified under the same screening criteria. Therefore, subsequent analyses were focused on the shared upregulated genes.

Heatmaps illustrate the expression patterns of these shared genes. As shown in Figure 4B, all five genes clearly exhibited higher expression levels in PDAC-affected tissues than in normal pancreatic tissues. Similarly, relative to non-diabetic individuals, increased expression of the same genes was evident in T2DM sufferers (Figure 4C). Thus, the concordant upregulation of a subset of genes in both PDAC and T2DM was demonstrated at the transcriptomic level.

To further contextualize these genes, we summarized previously published literature reporting associations between these genes and diabetes or PDAC (Table 1). All five genes—*FXYD3*, *LAMC1*, *MDFIC*, *SOCS3*, and *TREM2*—have been reported to be associated with diabetes and cancer-related conditions in prior studies.

Taken together, these findings demonstrate that PDAC and T2DM share a limited but distinct set of commonly upregulated genes, suggesting an overlap in the molecular alterations associated with the two disease conditions.

## 4. Discussion

In recent years, increasing epidemiological and clinical evidence has pointed to a close and complex relationship between PDAC and diabetes mellitus, and long-standing T2DM has been recognized as an important risk factor for PDAC. To explore this relationship, we performed an integrative analysis combining global epidemiological data and transcriptomic datasets. An analysis of GBD data revealed a continuous increase in global PDAC incidence and mortality due to PDAC from 2013 to 2023, which was accompanied by a parallel rise in the global incidence of diabetes (Figure 2) 22. These findings indicate a potential association between PDAC and diabetes; however, the observed parallel trends may be influenced by several confounding factors, including population aging, the rising prevalence of obesity, and improvements in diagnostic techniques. Therefore, the observed trends do not imply a direct causal relationship but rather highlight the need for further studies to clarify the underlying mechanisms. To further validate their epidemiological association, we performed a meta-analysis of eight previously published studies. Heterogeneity among studies was low (*I*² = 22.9%), and when the study results were pooled, diabetes was associated with a 1.94-fold increase in the risk of PDAC (95% CI: 1.78-2.11) (Figures 3). In addition, an analysis of gene expression profiles from two GEO datasets identified five genes—*FXYD3*, *LAMC1*, *MDFIC*, *SOCS3*, and *TREM2*—that were consistently upregulated in both PDAC and T2DM (Figures 3). Thus, convergent epidemiological and molecular evidence support a close association between diabetes and PDAC.

The five shared upregulated genes identified in this study may reflect convergent biological processes linking T2DM and PDAC, particularly in the context of metabolic dysregulation, inflammatory signaling, immune modulation, and tumor microenvironment remodeling. Among these genes, *FXYD3* is involved in the regulation of Na⁺/K⁺-ATPase activity and is predominantly expressed in epithelial cells. Its dysregulation may affect ion homeostasis and epithelial cell function, potentially contributing to both tumorigenesis and metabolic imbalance 23, 24. *LAMC1* encodes a major component of the basement membrane and is closely associated with extracellular matrix remodeling. In PDAC, this process is critical for tumor invasion and stromal interaction, while in diabetes, extracellular matrix alterations contribute to pancreatic fibrosis and tissue dysfunction. These findings suggest that *LAMC1* may serve as a structural link between the two disease states 25, 26. *MDFIC* a transcriptional regulator, may influence gene expression programs associated with cellular stress responses and metabolic adaptation, although its role in PDAC and diabetes requires further investigation 27, 28. *SOCS3* is a critical regulator of cytokine signaling and has been strongly implicated in insulin resistance through inhibition of insulin receptor signaling pathways. In the context of PDAC, *SOCS3* may contribute to a pro-inflammatory tumor microenvironment, thereby linking metabolic dysfunction with tumor progression 29, 30. *TREM2* is primarily expressed in myeloid cells such as macrophages, plays a key role in immune cell activation and modulation of the tumor microenvironment. Its upregulation may reflect enhanced immune remodeling and chronic inflammation observed in both diabetes and PDAC 31, 32. Taken together, these genes may converge on a common biological axis characterized by chronic inflammation, insulin resistance, immune activation, and tissue remodeling. However, it is important to note that these findings are based on bulk transcriptomic data and reflect associations rather than direct mechanistic evidence.

The shared upregulation of genes related to metabolic regulation, inflammatory signaling, and immune modulation has previously been suggested to represent potential biological pathways linking diabetes and PDAC 33. Chronic hyperglycemia and insulin resistance, hallmark features of T2DM, are known to promote a pro-inflammatory microenvironment and alter cellular metabolic states. Persistent low-grade inflammation and dysregulated cytokine signaling may, in turn, influence pancreatic tissue homeostasis and create conditions conducive to malignant transformation 34. In addition, diabetes-associated immune alterations, including changes in macrophage activation and immune surveillance, may contribute to tumor-promoting inflammatory responses within the pancreatic microenvironment 35, 36. Together, these metabolic and inflammatory disturbances may facilitate tumor initiation and progression, providing a plausible biological framework for the observed association between diabetes and PDAC.

This study has some limitations. First, as our analysis was based on publicly available data, the results are subject to the quality and heterogeneity of the original datasets. GBD database does not distinguish histological subtypes of pancreatic cancer. However, since PDAC represents the predominant subtype, accounting for over 90% of cases, the use of pancreatic cancer data as a proxy is generally considered reasonable. Nevertheless, this approach may introduce some bias and should be interpreted with caution 37, 38. Second, our transcriptomic analysis relied on bulk pancreatic tissue gene expression data and may not fully capture cell type-specific changes in the pancreatic microenvironment. Although both datasets were derived from pancreatic tissue, differences in cellular composition such as the presence of tumor epithelial cells, stromal fibroblasts, and infiltrating immune cells in PDAC samples, as well as varying degrees of islet dysfunction and inflammatory infiltration in T2DM may influence gene expression profiles. Such heterogeneity may confound the identification of shared differentially expressed genes and limit the direct comparability between datasets. In the present study, we attempted to mitigate this issue by performing independent analyses for each dataset and focusing on overlapping genes rather than directly integrating expression matrices. However, this limitation cannot be fully resolved in bulk transcriptomic analyses. Future studies using single-cell RNA sequencing or spatial transcriptomics may help to better delineate cell-type-specific mechanisms underlying the association between PDAC and diabetes. Third, the observational nature of the epidemiological analysis and the cross-sectional design of the transcriptomic dataset limit our ability to infer a causal relationship between diabetes and PDAC. Future research should include prospective cohort data, single-cell transcriptomic analyses, and experimental validation to further elucidate the biological mechanisms linking diabetes and PDAC and evaluate the clinical relevance of the upregulated genes common to both diseases identified in this study.

## 5. Conclusion

In summary, this study evaluated the relationship between PDAC and diabetes mellitus by integrating global epidemiological and transcriptomic data. Parallel increases in PDAC incidence and PDAC-related mortality, alongside a rising incidence of diabetes, highlight a concerning increase in global disease burden. Moreover, our meta-analysis demonstrated that diabetes was significantly associated with an increased risk of PDAC (pooled relative risk = 1.94, 95% CI: 1.78-2.11), strengthening the epidemiological evidence linking the two conditions. We identified five shared upregulated genes—*FXYD3*, *LAMC1*, *MDFIC*, *SOCS3*, and *TREM2*—which suggests that specific molecular alterations are associated with both conditions. Together, these findings support the existence of a close association between diabetes and PDAC at both the population and molecular levels and offer insights that may inform future risk stratification and mechanistic studies. Further investigations are warranted to validate these findings and clarify their clinical relevance.

## Supplementary Material

Supplementary tables.

## Figures and Tables

**Table 1:** Genes upregulated in both PDAC and T2DM, identified through GBD dataset analysis, along with the general and disease-related functions identified in previous studies reporting associations between the genes and diabetes mellitus or cancer.

Gene	Function	Related diseases	References
*FXYD3*	Associated with β-cell dysfunction in diabetes and promotes proliferation and progression in PDAC.	Diabetes, PDAC	39, 40
*LAMC1*	Extracellular matrix component; involved in islet microenvironment alteration in diabetes and enhances invasion and metastasis in PDAC.	Diabetes, PDAC	26, 41
*MDFIC*	Transcriptional regulator; linked to metabolic/inflammatory signaling in diabetes and contributes to tumor progression in PDAC.	Diabetes, colorectal cancer, PDAC	42, 27
*SOCS3*	Negative regulator of cytokine signaling; mediates insulin resistance and β-cell dysfunction in diabetes and modulates inflammation-related tumor progression in PDAC.	Diabetes, cancer	43, 44
*TREM2*	Immune receptor; regulates inflammatory responses in diabetes and shapes the immunosuppressive tumor microenvironment in PDAC.	Diabetes, PDAC	45, 46

Abbreviations include PDAC (pancreatic ductal adenocarcinoma), *FXYD3* (FXYD domain containing ion transport regulator 3), *LAMC1* (laminin subunit gamma 1), *MDFIC* (MyoD family inhibitor domain containing), *SOCS3* (suppressor of cytokine signaling 3), and *TREM2* (triggering receptor expressed on myeloid cells 2). References included in Table were selected through a targeted literature review focusing on studies reporting functional or disease related associations of the identified shared genes with PDAC, diabetes, inflammation, or tumor-related processes.

## Data Availability

Data from the manuscript are available from the corresponding author.
