# Supplementary material for: Epidemiological Trends and Shared Molecular Signatures of Pancreatic Ductal Adenocarcinoma and Diabetes: An Integrative Analysis Based on Global Burden of Disease and Gene Expression Omnibus Datasets
Source: J Cancer. 2026 May 25;17(6):1125–33. doi: 10.7150/jca.134528 (PMC13280538; doi:10.7150/jca.134528)
Supplement: Supplementary file 1 — Supplementary tables. [file jcav17p1125s1.pdf]

**Supplement Table 1. Characteristics of GEO datasets used in this study.**

| Dataset ID | Disease | Sample Size (Case/Control) | Tissue Source | Platform (GPL) | Clinical Information | Normalization Method | Batch Effect |
|---|---|---|---|---|---|---|---|
| GSE 15471 | PDAC | 36 / 36 | Pancreatic tissue (tumor vs adjacent normal) | GPL570 (Affymetrix Human Genome U133 Plus 2.0 Array) | Limited (tumor vs normal status available) | RMA + log2 transformation | Not required |
| GSE 20966 | T2DM | 10 / 10 | Human pancreatic islets | GPL1352 (Affymetrix Human X3P Array) | Limited (diabetes status available) | RMA + log2 transformation | Not required |
